# Coupling development of sports industry and tourism industry based on internet of things

**DOI:** 10.1371/journal.pone.0299080

**Published:** 2024-04-18

**Authors:** Tongchang Hu, Xi Wang, Quantao He, Jinbo Bei

**Affiliations:** 1 Football Academic, Wuhan Sports University, Wuhan, Hubei, China; 2 School of Economics and Management, Shanghai University of Sport, Shanghai, China; 3 Sport School, Shenzhen University, Shenzhen, China; 4 Graduate School, Guangzhou Sports University, Guangzhou, Guangdong, China; Hungarian University of Agriculture and Life Sciences: Magyar Agrar- es Elettudomanyi Egyetem, HUNGARY

## Abstract

This study investigates the positive coupling between the sports industry and tourism, exploring the ways to promote their interconnection. Under state guidance, the integration of sports industry services is facilitated to attract sports culture and tourism fairs, leveraging regional economic development advantages to enhance the industrial market appeal. The emerging leisure consumption mode of sports tourism injects vitality into the economy, fostering the core sports service industry. The coupling of the education and tourism sectors is strategically aligned with long-term national policies. Using IoT technology, this paper employs a grey relational analysis to assess the coupling between the sports industry and tourism, revealing a significant correlation. Experimental results demonstrate a positive coupling trend, likened to conjoined twins with a natural material basis and technical support. This coupling not only aligns with industry trends but also resonates with the "environmental protection era," "green era," and "ecological era," marking a pivotal aspect of industrial development. The study contributes valuable insights into the symbiotic relationship between the sports and tourism industries, emphasizing their interconnectedness and the positive implications for economic and environmental sustainability.

## 1. Introduction

The sports industry has emerged as a focal point of global interest, mirroring the rapid expansion of the modern worldwide society and economy. Notably, China’s sports sector has experienced substantial growth, aligning with government initiatives and providing a promising avenue for ongoing development. An intriguing prospect arises as the tourism and sports sectors collaborate synergistically to further these goals in the long term. This strategic alliance reflects a forward-thinking approach. The interplay between sports and tourism holds immense potential to expand the industry’s horizons, reshape its organizational structure, and stimulate increased consumption within China’s sports market [1].

In the current situation, it becomes necessary to explore cooperative growth strategies between the sports and tourist businesses. By utilizing the combined assets of the two industries, this multidisciplinary strategy aims to establish a symbiotic connection that improves economic viability while also enhancing the general cultural and recreational landscape [2]. Sports and tourism integration supports the overarching objectives of national policies by highlighting the necessity of all-encompassing, cross-sectoral actions [3]. The capacity of this collaborative development approach to take use of cutting-edge industry platforms that promote interactive and mutually beneficial growth is critical to its success. Utilizing cutting-edge technology, digital platforms, and data-driven insights becomes crucial for expanding the organic business outside its conventional boundaries. In addition to improving the efficacy and efficiency of sports-related activities, these cutting-edge solutions open up new possibilities for audience engagement, tourism experience optimization, and the development of a vibrant ecosystem [4].

One of the most important aspects of the industrial sector’s value addition is the industrial coupling’s developmental trajectory, which is a dynamic process [5]. This unique phenomenon is different from industrial differentiation; it is a developmental paradigm that is taking place in the context of global economic integration and the fast expansion of high-tech industries. Industrial coupling functions through a dynamic interplay, as opposed to the conventional model of distinct and separate industries, wherein multiple independent sectors gradually blend at their edges, eroding the boundaries that were once clearly defined and ultimately giving rise to a more cohesive and integrated industrial landscape. This revolutionary technique optimizes the whole industrial structure in addition to fostering the creation of new businesses. It also greatly increases the potential for institutional, scientific, and technical innovation, which combines to provide a fresh nexus for economic growth [6].

This paper stands out due to its utilization of grey relational analysis to explore the Internet of Things (IoT) and its impact on discerning the coupling relationship between the sports and tourism industries. Employing this analytical framework offers a nuanced comprehension of the complex interconnections and interdependencies existing between these two sectors. By revealing the subtle intricacies of their relationship, the paper not only imparts valuable insights into the dynamics of industrial coupling but also suggests specific measures to promote the mutually advantageous growth of both the sports and tourism industries.

## 2. Literature review

People’s higher level demands are enhanced after their physiological level wants are met. People set higher criteria for life quality as the standard of living in their communities rises. People are becoming more and more interested in participating in sports as economic system reform intensifies. Naidoo K thought that the athletic goods sector had grown quickly in recent years. There was a big market demand, but the industry was still poorly regulated [7]. Funk studied how to strengthen the consumption desire of sports consumers and determine the future trends of the sports industry [8]. Wood L believed that the sports industry had changed a lot in the past decade. New technologies, public policies and changing customer expectations have all played and continue to play a role in industry transformation. It is technology coupled with the right policy and regulatory support that provides the necessary ingredients to continue driving the transformation of the industry [9]. Hsieh L Y believed that it was crucial for regional and national tourism businesses to respond to the current economic crisis by boosting profits during the recession. He discussed the benefits of lowering the level of tourism hotel landscape service quality on improving profits [10]. Taking the tourism industry as an example, Dimitrios B discussed the historical evolution of knowledge and the reasons why knowledge had become an influential factor in an organization’s hope of survival. He also discussed how knowledge management could be a useful tool for leveraging tourism organizations [11]. Scholars believed that both the sports industry and the tourism industry could drive people’s economic development and increase employment opportunities. If the two industries are coupled, it would bring greater effect. However, they did not explain how to couple the two industries.

The Internet of Things has been developed at an increasingly rapid rate in recent years, which has enormous significance for humans. According to Perera C, the Internet of Things (IoT) is a dynamic global information network made up of Internet-connected items like smart appliances that are quickly taking over as a major component of the Internet of the future. He extensively examined more than 100 IoT smart solutions available on the market [12]. Mostafa H thought that the current focus of practically all IoT-related conversations was on wearables. The number of older persons was projected to significantly rise by 2020, which increased the demand for health monitoring and preventive medicine. The overall cost of prevention and surveillance could be decreased with the help of advanced technology [13]. Wireless sensor networks, according to Aranzazu-Suescun C, had a wide range of uses, including smart homes, smart cities, fire detection systems, and climate monitoring systems. Wireless sensor networks were crucial for gathering environmental data and for monitoring and reporting the environment [14]. IoT, according to Mosenia A, was viewed as a revolutionary method of delivering a wide range of services. The Internet of Things relies heavily on compact smart devices, which come in a variety of applications, sizes, energy capabilities, and computational capability. However, there are various security issues that come up with the incorporation of these smart gadgets into the mainstream Internet [15]. According to experts, the Internet of Things has greatly improved people’s quality of life.

The recent literature [16–20] demonstrates the growing interest in the intersection of the sports and tourism sectors, especially in light of emerging technologies like the Internet of Things (IoT). Nevertheless, a thorough review of the literature identifies a significant lack of explanation for the complex dynamics and dynamic nature of the coupling connection between these sectors. While prior research has acknowledged the potential synergies, further in-depth investigation is needed to fully understand the precise processes and effects of their integration, particularly with regard to the use of IoT technologies. By using gray relational analysis to elucidate the complex relationships between the sports and tourist industries, this study aims to close this gap. Through an exploration of the dynamic terrain molded by industrial coupling and the function of IoT, this research attempts to offer a thorough grasp of the interactions among these sectors. Furthermore, the study aims to provide tactical approaches to promote their mutually beneficial development, which will add significant value to the developing conversation about the intersection of sports, tourism, and technology.

## 3. Materials and methods

**Fig 1:**
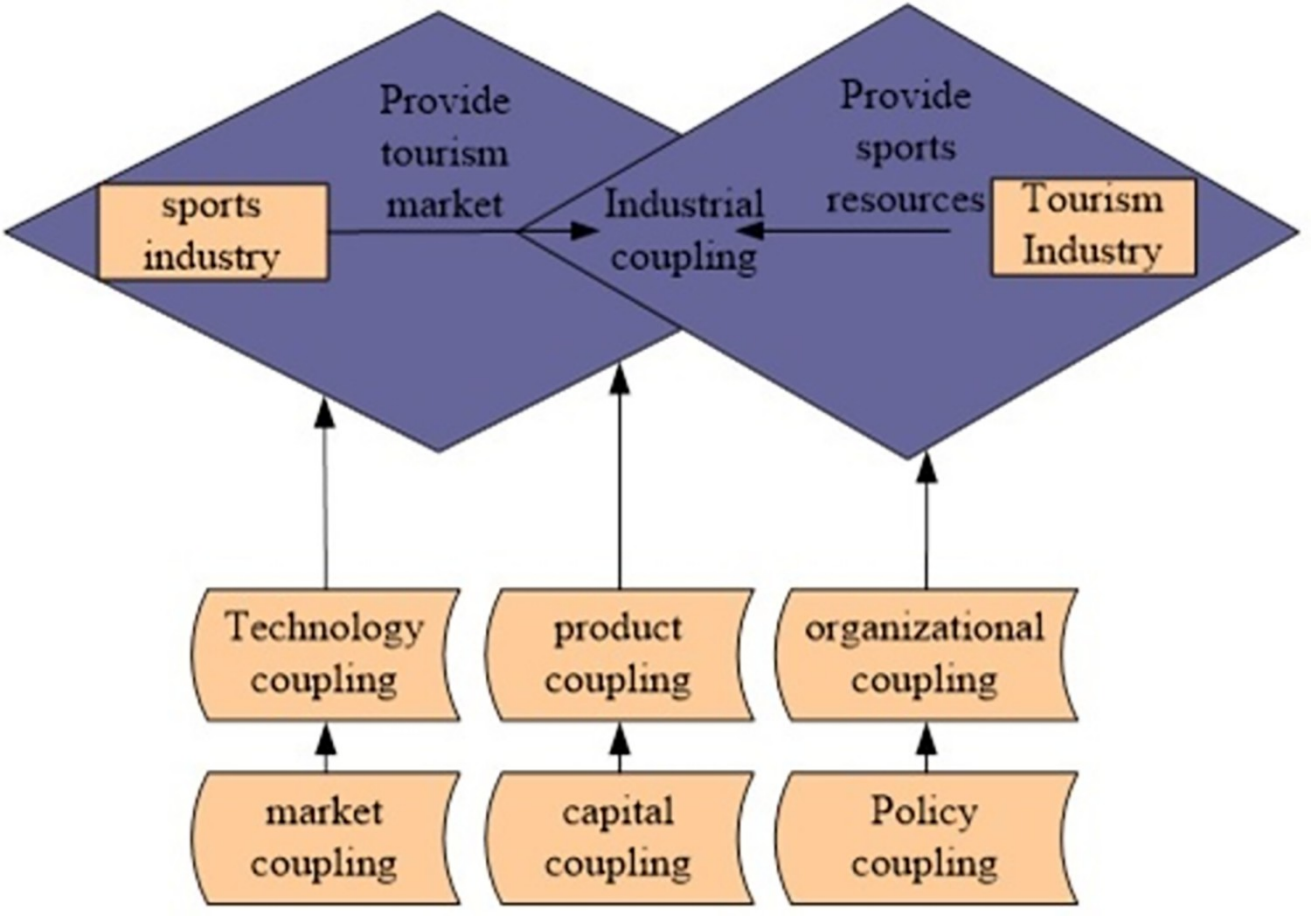
Dynamic coupling mechanism diagram of tourism industry and sports industry.

Due to the intangibility and uncertainty of sports products, consumers cannot detect the quality of sports products in advance. This leads to information asymmetry between product suppliers and consumers, which affects the sustainable development of the sports industry. The monopoly of the sports industry market makes consumers more likely to buy low-quality sports services at high prices [21]. Therefore, it is necessary to improve the service quality through the service providing platform, so that consumers can experience sports while providing more appropriate services. Through docking with related industries, sports services can be combined with other services to obtain additional benefits to better meet the needs of consumers. The coupling between sports industry and tourism mainly includes technology coupling, product coupling, market coupling, capital coupling and political coupling. The coupling status of sports industry and tourism industry is shown in Fig 1.

As shown in Fig 1, major sports events, large venues and facilities, fitness routes and other sports resources are used as production factors to provide a strong impetus for the development of tourism. The sports and tourism industries use these resources as the basis for mutual development. While utilizing and developing sports resources, it can not only be effectively utilized, but also become a tourist attraction and attract more tourists, which supports local economic development and ensures mutual benefit and win-win between the sports industry and tourism [22].

With the development of modern science, many new fields appear. It aims to reveal the deeper interconnectedness of things, solving long-standing problems in many fields of science. Based on the highly differentiated nature of modern scientific research, a new set of disciplines with methodological significance—grey correlation analysis has begun to appear [23]. The emergence of grey relational analysis not only solves some long-standing problems, but also promotes the overall development of science and technology. It enables people to have a deeper understanding of the nature of the connection between things and the objective laws of their development.

### 3.1 Grey relational model

**Fig 2:**
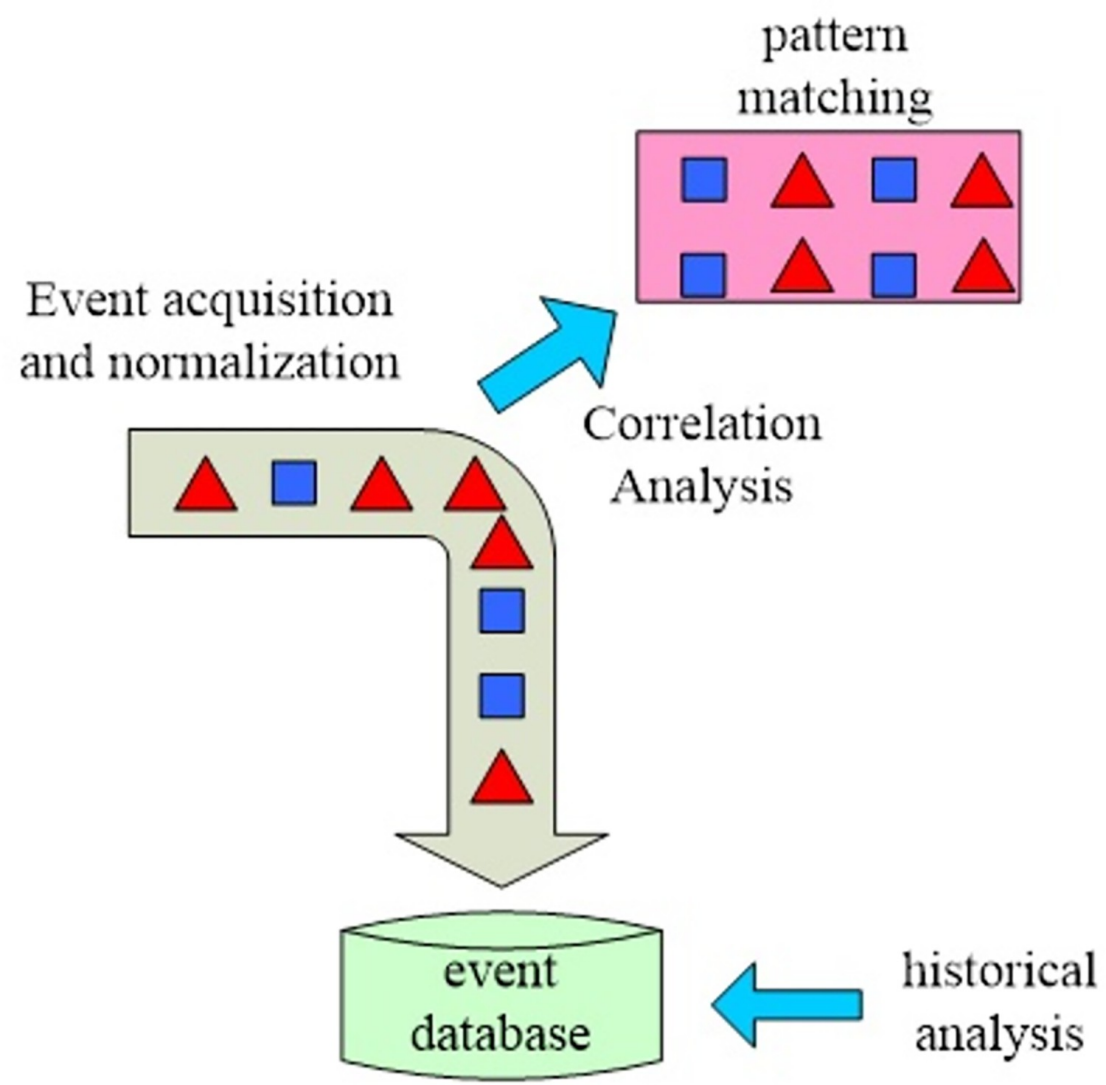
Grey relational analysis.

One of the main advantages of grey relational analysis is the high plasticity of the data, which does not require large samples or complete information. Even if the data is unordered, it does not affect the results of quantitative analysis [24]. The grey relational analysis is shown in Fig 2.

As shown in Fig 2, grey relational analysis has been widely used in many fields due to its high plasticity, which has successfully solved many practical problems and achieved good application results [25]. Grey relational analysis is a quantitative analysis of the dynamic development of system factors. The first step in creating a grey relational analysis is to decide which series to analyze. The s subfactors of the reference sequence containing system are expressed as Formula (1):

$$B_{j}(k)(j=1,2,\ldots ,s;k=1,2,\ldots ,n)$$ (1)

Next, the comparison sequence is determined, and the comparison sequence contains m sub-factors that have an impact on the system, denoted as Formula (2):

$$A_{j}(k)(j=1,2,\ldots ,m;k=1,2,\ldots ,n)$$ (2)

Due to the large differences in the indicators, direct comparison and analysis cannot be carried out, so accurate and reliable conclusions cannot be drawn. To make the data comparable, the raw data have been transformed into comparable series through dimensionless processing. The mean transformation uses the mean method for dimensionless data, as it can visualize the growing and decreasing trend of the data [26], as Formulas (3) and (4):

$$B_{j}{\left(k\right)}^{0}=\frac{B_{j}(k)}{\bar{B}},k=0,1,2,\ldots ,n$$ (3)

$$A_{i}{\left(k\right)}^{0}=\frac{A_{i}(k)}{\bar{A}},k=0,1,2,\ldots ,n$$ (4)

The absolute difference between the comparison sequence and the reference sequence is found, and then the maximum and minimum differences are found [27]. The absolute difference between the values of the comparison series and the reference series is Formula (5):

$$\Delta_{i}(k)=|A_{i}{\left(k\right)}^{0}-B_{j}{\left(k\right)}^{0}|,k=0,1,2,\ldots ,n$$ (5)

The maximum and minimum ranges are:

$$M=\max_{i}\max_{k}\Delta_{i}(k)$$ (6)

$$m=\min_{i}\min_{k}\Delta_{i}(k)$$ (7)

The coupling number of the factor of the comparison sequence and the corresponding factor of the reference sequence is Formula (8):

$$\xi_{i}(k)=\frac{m+\rho M}{\Delta_{i}(k)+\rho M},k=1,2,\ldots ,n$$ (8)

*ξ*_*i*_(*k*) represents the gray-off coupling number, and the larger the value, the stronger the correlation at time k. *ρ* represents the resolution factor.

A correlation matrix consisting of average coupling numbers is created to reflect the interrelationship between the coupling effects of the two branches [28]. The grey correlation matrix is constructed as Formula (9):

$$\tau =(\gamma_{ij})=[\begin{array}{cccc} \gamma_{11} & \gamma_{12} & \ldots & \gamma_{1m}\\ \gamma_{21} & \gamma_{22} & \ldots & \gamma_{2m}\\ \ldots & \ldots & \ldots & \ldots \\ \gamma_{s1} & \gamma_{s2} & \ldots & \gamma_{sm} \end{array}]$$ (9)

This paper examines the interaction mechanism between the sports industry and the tourism industry from a macro perspective. The grey model is used to analyze the interaction force between the two industries to further explore the internal driving force and interaction force of the coupled development of the two industries [29]. The grey correlation model is used to examine the correlation strength between the factors of the indicators, and then the dominant factors of the interaction between the two industries are determined.

### 3.2 Grey correlation degree and model of coupling between sports industry and tourism industry

The sports and tourism industries together form a complex grey system. Grey model is a quantitative research technique, which uses the relevant methods of grey system theory to test the coordinated development of the two. This model can be used to study the internal organization of the system, as well as the number and structural interactions of elements on different time scales [30]. Known data in a grey system is often scattered. In order to establish differential formulas, this gray sequence must be transformed into a more regular sequence, and this approximation is called gray differential formula model in gray system theory [31].

According to Formula (10), it is assumed that the system has N behavioral factors.


$$a_{i}^{\left(0\right)}=(a_{i}^{\left(0\right)}(1),a_{i}^{\left(0\right)}(2),\ldots ,a_{i}^{\left(0\right)}(n))$$ (10)

Among them, $a_{i}^{\left(0\right)}$ is used as a system-related factor variable, and $a_{i}^{\left(0\right)}(n)$ is used for accumulation operation, and an accumulation generated sequence can be obtained as Formula (11):

$$a_{i}^{\left(1\right)}=(a_{i}^{\left(1\right)}(1),a_{i}^{\left(1\right)}(2),\ldots ,a_{i}^{\left(1\right)}(n)),\;i=1,2,\ldots ,N$$ (11)

Differential formulas are transformed into discrete models:

$$d_{1}(k)+a_{1}z_{1}^{\left(1\right)}(k)=a_{2}z_{2}^{\left(1\right)}(k)+a_{3}z_{3}^{\left(1\right)}(k)+\ldots +a_{N}z_{N}^{\left(1\right)}(k)$$ (12)

These coefficients can be used to describe the overall industry level, correlation and dynamics of individual industry factors. They represent the ability of major behavioral variables to evolve in the system and play a decisive role in current and future development. The control coefficient is a descriptive parameter of the effect of each factor on the main behavioral variable.

The least squares method is usually used to find an estimate of a, which means that when $a_{N}z_{N}^{\left(1\right)}(k)$ is invertible, there is Formula (13):

$$\overset{\wedge }{a}={\left(A^{T}A\right)}^{-1}A^{T}D$$ (13)

When *a*≻*a*_1_ is satisfied, that is to say, this quantity increases in quantity when the system has the ability to develop. The larger it is, the stronger its internal development ability is.

A gray model is used to illustrate the degree of development of the sports sector and the degree of development of the tourism business:

$$A_{1}^{\left(0\right)}=\{A_{1}^{\left(1\right)}(1),A_{1}^{\left(1\right)}(2),\ldots ,A_{1}^{\left(1\right)}(10)\}$$ (14)

The relevant comparable variable is the development level of the tourism sector, and the development level of the sports industry is the primary variable. The mechanism of the sports industry’s influence on the tourism industry is examined as follows:

$$z_{1}^{\left(1\right)}(k)=\frac{1}{2}(a_{1}^{\left(1\right)}(k-1)+a_{1}^{\left(1\right)}(k))$$ (15)

The sports industry’s mechanism analysis is conducted on the tourism sector using the same methodology as described above. The major system behavior variable in the grey model is the level of development of the sports industry. The relevant factor variable is the tourism sector:

$$a_{2}^{\left(1\right)}(t)=\frac{da_{1}^{\left(1\right)}(t)}{dt}-a_{1}^{\left(1\right)}(t)$$ (16)

The relationship between the sports and tourism industries demonstrates the tourism sector’s capacity for both past and present self-development and sustainable growth. The sports sector has contributed to the promotion of tourism. Another key factor influencing the growth of the sports industry is tourism. The aforementioned findings demonstrate that both the sports business and the tourism industry have strong capacities for self-development, which are on the rise. It is clear that the expansion of the sports industry is largely a result of the tourism sector.

Sports and tourism are connected industries that impact and interact with each other constantly as they grow. The degree of coordination, often referred to as the degree of coupling correlation, is a measure of the distance between static systems, which determines whether the systems are coupled or not.

In this case, T represents the sports industry and R represents tourism. The smaller the c is, the better it is, then:

$$c=\frac{2|F_{1}(a,t)-F_{2}(b,t)|}{\left(F_{1}(a,t)+F_{2}(b,t)\right)}$$ (17)

The relationship between the sports industry and the tourism business can be defined by using the formula as follows:

$$c_{l}=\frac{2\sqrt{F_{1}(a,t)•F_{2}(a,t)}}{F_{1}(a,t)+F_{2}(a,t)}$$ (18)

*c*_*l*_ is obtained by squaring *c*_*l*1_. If 0≤*c*_*l*1_≤1, it demonstrates how crucial the connection between the sports business and tourism is. Consequently, to determine the degree of association between the sports business and tourism, *c*_*l*_ can be used as the measurement model.

The association between tourism and the sports business can be evaluated using *c*_*l*_. The model, however, cannot always accurately represent the state and level of the two industries in some particular circumstances. The ultimate goal of evaluating the importance of the tourist and sports industries is to encourage mutually beneficial growth between them. If only *c*_*l*_ is used to measure the correlation, the results obtained are not scientific and instructive.

It is vital to build a coordinating model for the interaction of these two businesses in order to reflect the reality of tourism and sports, as this is the only method to reflect the growth of these two industries. The indicator used to quantify the degree of growth in the correlation between two industries is referred to as the degree of coupling in this article.

$$D={\left(c_{l}•T\right)}^{\varsigma }$$ (19)

$$T=\alpha F_{1}(a,t)+\beta F_{2}(b,t)$$ (20)

*c*_*l*_ stands for relevance. Despite being very straightforward, the combined coordination degree model incorporates the coordination state D of the travel and sports sectors. It also shows the T-level of development for the two industries, which can keep their levels from being too low. The model contains the traits of integration, simplicity, and lack of harshness, but the coordination of the two industries is subject to some unique conditions. This model, which may be used to assess the development of the tourism and sports industries in the same region or in separate regions, has a wider range of applications and is more stable than previous models.

### 3.3 Internal coupling between sports industry and tourism industry

The strong relationship between the sports and tourism industries can hasten the growth of sports tourism. Additionally, it can offer incentives for the growth of the sports and tourist industries, which strengthens the development of sports tourism. Sports tourism may take longer to grow due to the weak links between the sports business and tourism. Not only the positive effects of their coupled development are not obtained, but also the negative effects of their coupled development appear.

(1) Development of tourism provides hardware support for the development of sports

**Fig 3:**
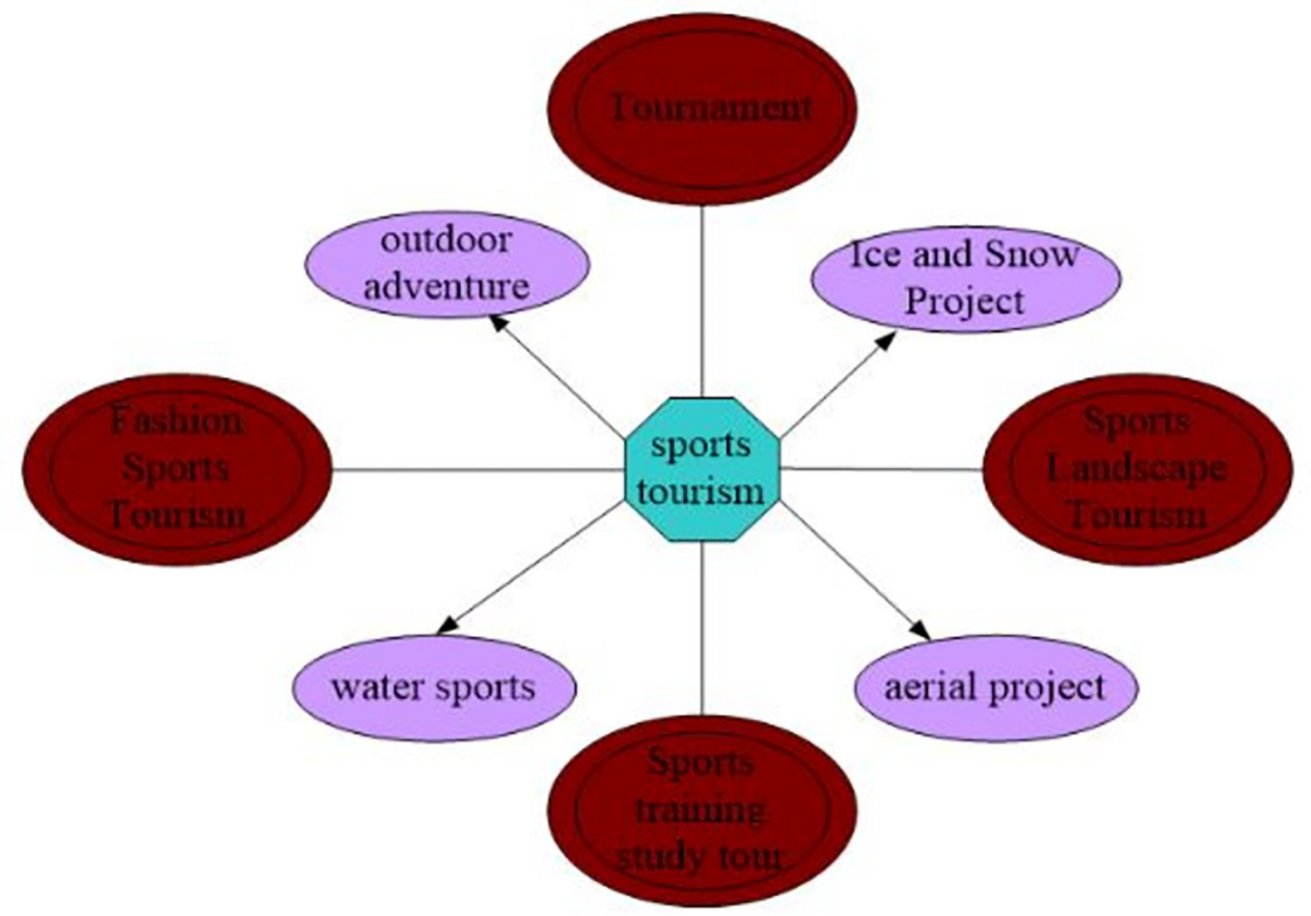
The association between sports and tourism.

Some tourist attractions must meet the needs of tourists in terms of food, accommodation and transportation. Tourism has led to the development of various local facilities, including transportation, hotels and restaurants, creating conditions for the development of the sports industry. This is especially applicable to areas where clustering effects can be rapidly generated due to the organization of important activities, which places very high demands on city management and equipment, as shown in Fig 3.

As shown in Fig 3, the rapid development of tourism has created conditions for the development of sports activities. When bidding for the Olympic Games, the Olympic Committee conducted a comprehensive assessment of the city, including the city’s management level and infrastructure level. The bidding city’s environment, transportation and accommodation were also evaluated. These facilities are closely related to the development of tourism. With the rapid development of the tourism industry, the tourism industry has reached a perfect state, which in turn promotes the development of the sports industry and provides opportunities for some cities to hold important events.

(2) Tourism development provides a source of tourists for sports development

**Fig 4:**
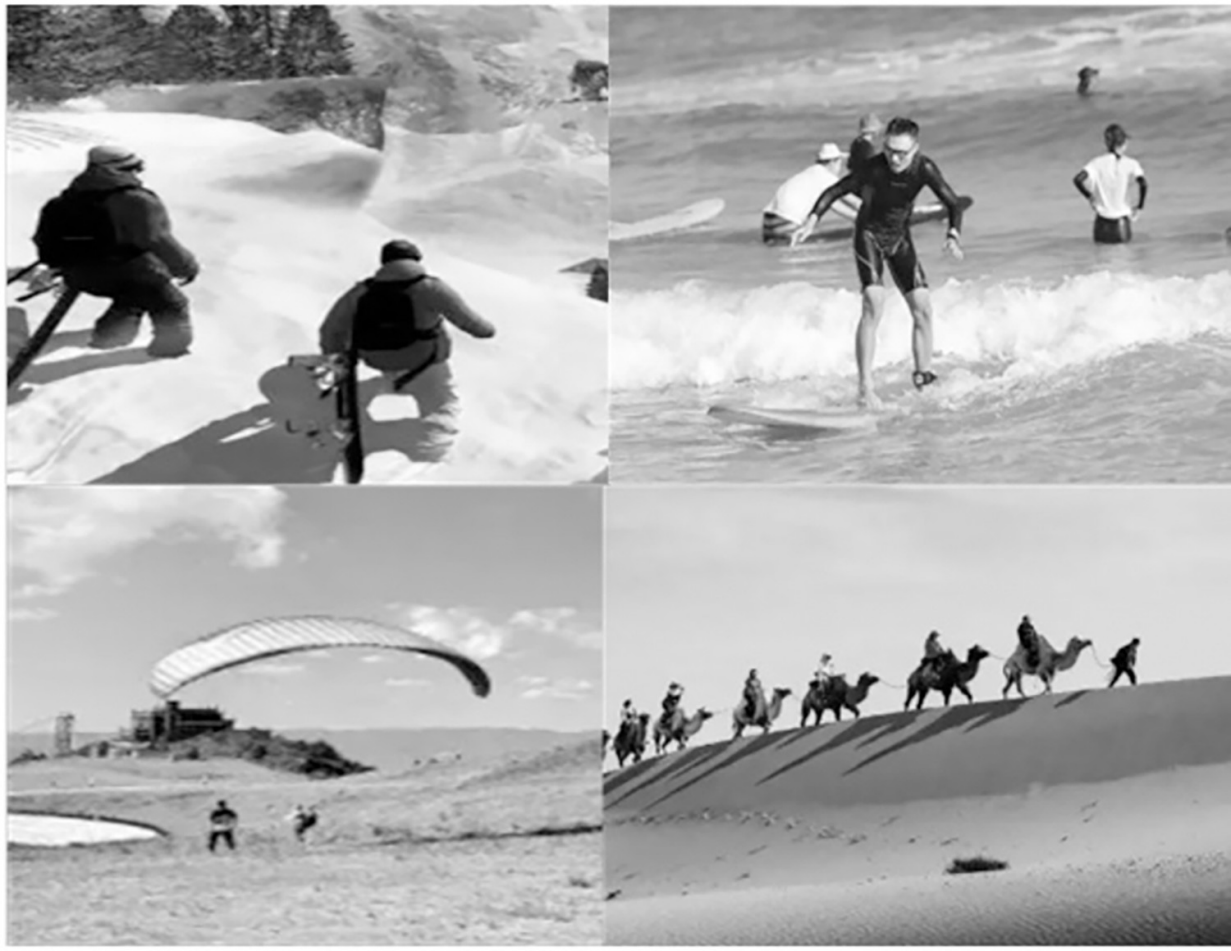
Tourism project.

Some cities are able to attract tourists in the process of developing tourism, which encourages sports activities with people. Since entering the new century, China has paid more attention to the development of tourism and actively cultivated new tourism markets. In 2010, there were more than 2.1 billion Chinese tourists, more than 130 million international tourists, and more than 55 million international overnight tourists. The tourism project is shown in Fig 4.

As shown in Fig 4, the development of sporting events is based on the arrival of these tourists. With the rapid development of tourism, more potential tourists need features, quality and content. If a city hosting a sporting event has a booming tourism industry and various tourism-related resources, tourists are attracted to the city to participate in the city’s resources, and the city can profit from it.

The challenge of how to disperse the benefits arises when the sports and tourism industries are coupled, which has an impact on how the coupled system of the sports and tourism industries develops. The establishment of a just and scientific benefit-sharing mechanism is required to realize the coupled system’s sustainable development and ensure its continued growth.

## 4. Results and discussion

### 4.1 Development status of China’s tourism industry

**Fig 5:**
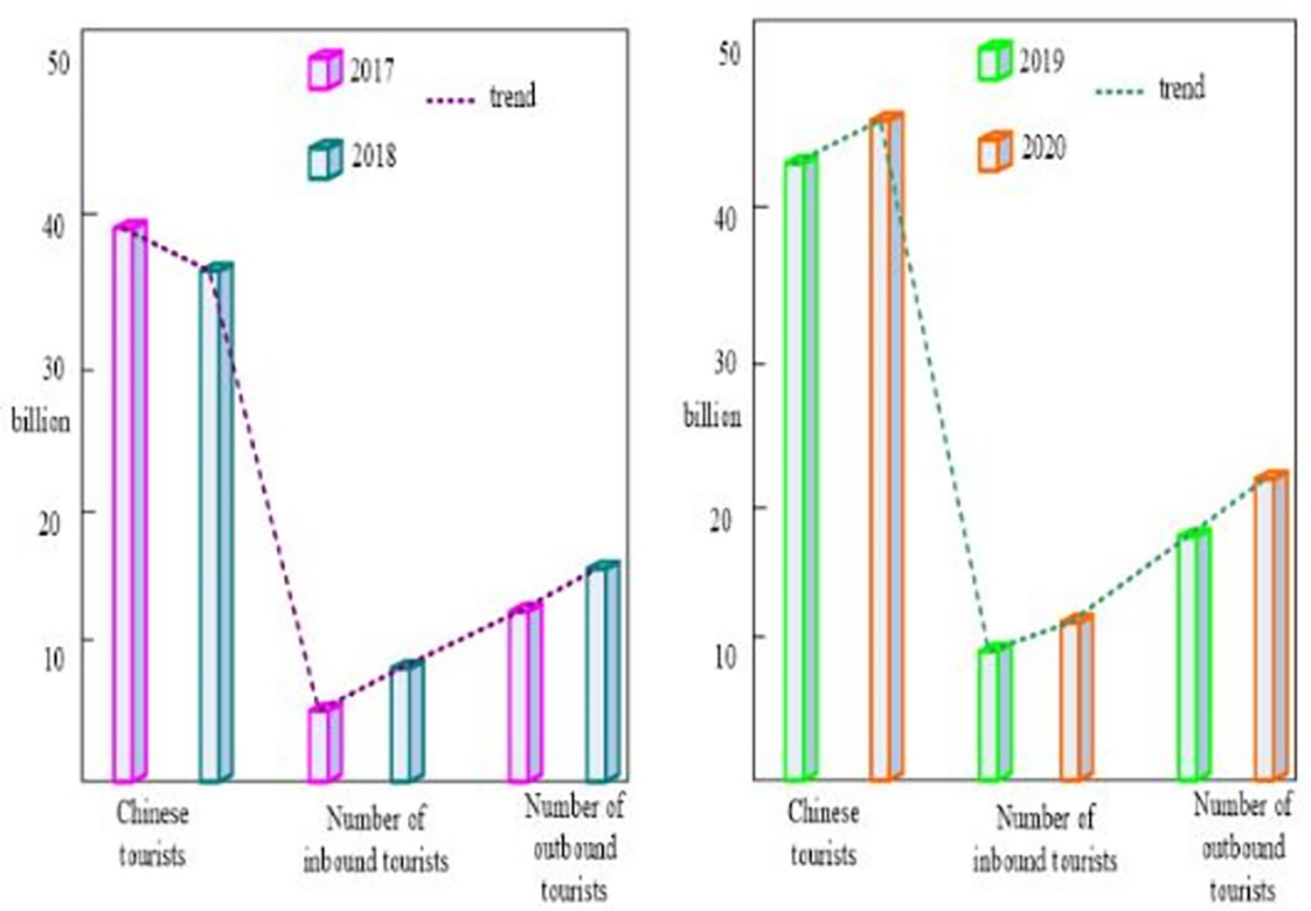
Tourism industry development from 2017 to 2020.

Tourism has strong regional characteristics. Taking the regional space as an example, when the content of the regional space changes, the tourism industry also changes. Since tourism has no clear boundaries, in a sense it is more inclusive and malleable in space. Its own value is that it can link the region with other industries to obtain economic benefits [32]. Since the reform and opening up, especially since the "Twelfth Five-Year Plan", China’s tourism industry has developed rapidly and has a wide coverage. The development of the tourism industry from 2017 to 2020 is shown in Fig 5.

As shown in Fig 5, tourism is developing rapidly in some regions. From the perspective of tourism development, it is obviously developed on the basis of a good environment and a good atmosphere. While tourism shares certain similarities with other industries, it also has a unique quality, which is that it relies heavily on local brands. In fact, if a local resource can provide space or content for tourism, it can become a tourism sector. However, in order to ensure that the tourism industry contains tourism characteristics and content and enhance the competitiveness of tourism, it must be based on strong communication channels.

### 4.2 Development status of China’s sports industry

**Fig 6:**
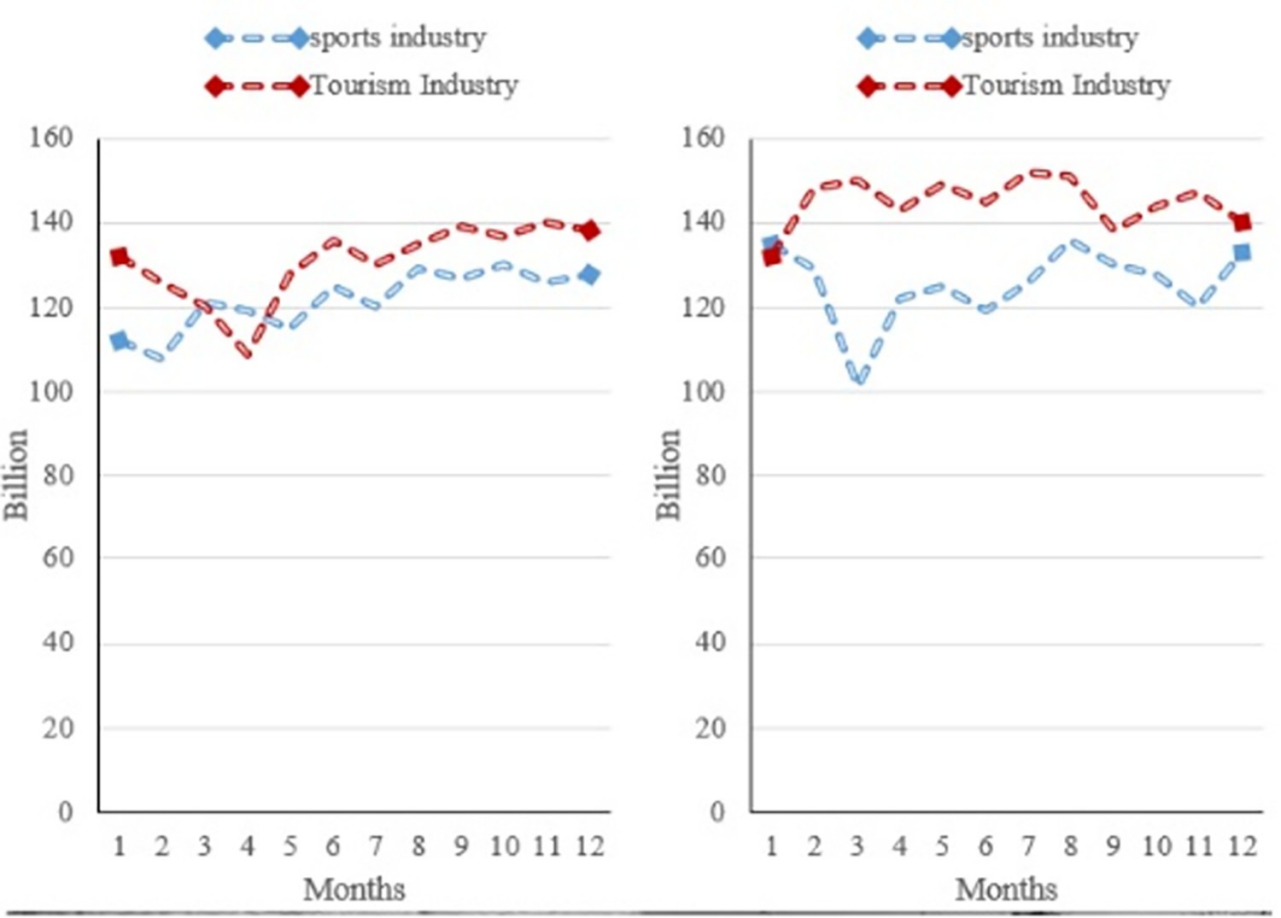
The sports industry and the driving tourism industry from 2017 to 2018. (a) 2017 sports industry and driving tourism industry. (b) 2018 sports industry and driving tourism industry.

The quality of life of the populace has significantly increased in recent years as a result of the acceleration of economic and social growth. Sports are progressively becoming more industrialized and extending into other spheres. It is connected to other sectors of the same value chain. The sports industry is quickly growing as the economic value of sport becomes increasingly clear. The Chinese economy has consistently benefited from the sports sector’s significant contribution, which has increased employment prospects for the populace and sped up the growth of allied industries. Fig 6 depicts how the sports industry changed between 2017 and 2018.

When analyzing the added value of the sports business from 2017 to 2018, a more detailed analysis shows differences in the growth paths of the various sports industry sectors, as Fig 6’s data demonstrates. A thorough analysis of this comparison is given in Fig 6(A), which shows a steady increase in the added value produced by the sports industry. In particular, in 2017 the sports sector’s value contributed surpassed a significant benchmark—that is, $10 billion USD. The next image, Fig 6(B), illustrates a further progression in the sports industry’s added value in 2018, with a notable rise to more than $12 billion USD. The industry is continuing to grow positively, despite the relatively small percentage gain. This is seen in the overall trend. This slow increase reflects how people’s lives are changing and how their quality of life is improving at the same time. The discrepancies that have been noted and the increasing trend in added value highlight the diverse growth that various sports sector areas have had. This expansion shows the industry’s adaptation to shifting consumer habits and societal tastes in addition to demonstrating its durability. The ongoing rise in added value indicates a healthy development in the sports and tourist markets, pointing to a mutual reliance between these two industries. Most importantly, this evidence confirms the complex connection between enhanced quality of life, societal shifts, and the growing demand for sports-related activities. The need for more varied and stimulating recreational activities makes the sports sector a crucial engine of economic expansion. As a result, the tourist business, which is intimately linked to the sports sector, gains from these advancements and expands quickly in line with the increasing added value produced by the sports sector.

Together, China’s sports industry and related industries provide a substantial contribution to the sports industry, contributing more than 60% of the industry’s total added value. This significant contribution highlights how these associated businesses have shaped and advanced the overall growth of China’s sports industry. This significant contribution is closely related to China’s unique approach to sports management, which has a positive impact on the industry’s dynamics and growth. China’s approach to sports management, characterized by distinct regulations, tactics, and institutional frameworks, is essential in facilitating the cooperation of various sports industry players. This management approach has an influence on other industries that are a part of the larger value chain, in addition to the traditional confines of sports. The positive effect is seen in the sizeable portion of added value that is ascribed to these related industries, demonstrating the efficacy and flexibility of China’s sports management strategy. A thorough analysis of the added value that each industry in the sports sector contributes is given in Table 1. This table’s composition clarifies the several sectors that come together to make up China’s sports business. Sportswear, sporting products, stadiums, physical fitness, sports training, sports lotteries, and sports building are among the noteworthy sectors that make up this complex web. These categories demonstrate the diversity and expertise within the larger sports business, with each contributing a certain percentage to the overall added value. Sportswear stands out as one of these categories that contributes the most, accounting for 62.7% of the total added value. This emphasizes how important customer tastes and market demand are for sportswear. Sporting goods comes in second, with a 12.5% share, highlighting the significance of gear and accessories in the sports industry. The multidimensional character of the sports sector is shaped by the contributions of stadiums, physical fitness, sports training, sports lotteries, and sports building, each of which contributes in a unique way to the production of total value.

**Table 1:** Added value of China’s sports industry.

projects	Percentage%	effective percentage%
sportswear	62.7%	62.7%
sporting goods	12.5%	12.5%
stadiums	3.6%	3.6%
physical fitness	5.8%	5.8%
sports training	5.4%	5.4%
sports Lottery	5.1%	5.1%
sports building	4.9%	4.9%

As shown in Table 1, sportswear has the highest proportion of added value at 62.7%, and sporting goods has the second highest proportion at 12.5%. Stadiums is 3.6%, physical fitness is 5.8%, sports training is 5.4%, and sports lottery is 5.1%. There are significant differences in the level of development of the various branches of the sports industry. This phenomenon is caused by the link between the characteristics of individual industries and the way they operate. However, it also reflects the imbalance of China’s sports industry structure, which should be actively promoted to improve and perfect the structure.

### 4.3 Development status of sports tourism

As an emerging industry combining sports and tourism, sports tourism has no special planning and development and protection policies, and the publicity of sports tourism is not enough, which leads the masses to not pay attention to this work.

This paper surveys 350 students in a sports university to determine whether they understand sports tourism knowledge and the content of tourism activities. The survey results are shown in Table 2.

**Table 2:** Knowledge of sports tourism.

Learn degree	number of people	Percentage%	effective percentage%
know very well	110	31%	31%
better understanding	80	23%	23%
General understanding	75	21%	21%
don’t understand	50	14%	14%
very ignorant	35	11%	11%

As shown in Table 2, there are 110 people who are very aware of sports tourism, accounting for 31%. There are 80 people who are more aware of sports tourism, accounting for 23%. There are 75 people who generally know about sports tourism, accounting for 21%. The number of people who do not know about sports tourism is 50, accounting for 14%. There are 35 people who do not know much about sports tourism, accounting for 11%. It can be seen that most of the students still understand sports tourism, which indirectly shows that the sports industry and tourism industry are developing better.

**Fig 7:**
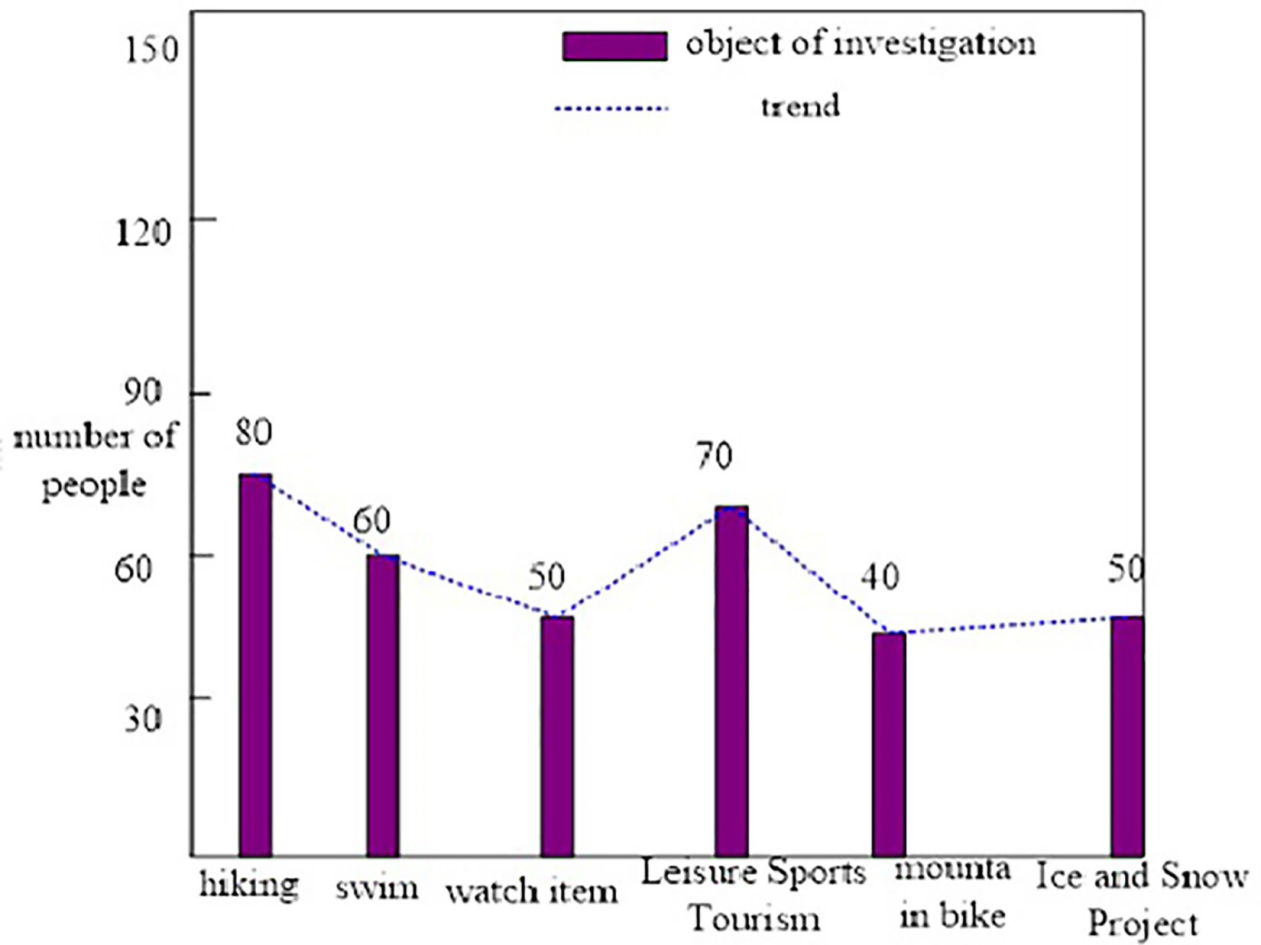
Sports events in the tourism industry.

The survey items in this paper are divided into hiking, swimming, watching or participating in sports events, leisure sports tourism, mountain biking, ice and snow events, etc., to understand the respondents’ participation in various activities during the travel process. The survey results are shown in Fig 7.

As shown in Fig 7, hiking, swim, watching or participating in sports events, leisure sports tourism, mountain biking, and ice and snow events are all popular. It is encouraged that the interaction between sports and tourism is gaining momentum. In particular, major sporting events have become the most important contributors to the tourism industry.

This paper can determine the degree of coordination between the tourism industry and the sports industry and its indicators, as shown in Table 3.

**Table 3:** Coupling coordination degree.

years	*F*_1_(*a*,*t*)	*F*_2_(*b*,*t*)	D	Evaluation
2014	0.715	0.815	0.890	well coordinated
2015	0.724	0.802	0.895	well coordinated
2016	0.729	0.823	0.903	Quality coordination
2017	0.736	0.826	0.905	Quality coordination
2018	0.738	0.829	0.917	Quality coordination
2019	0.742	0.835	0.926	Quality coordination

The tourism and sports sectors in China have been intertwined since China’s early development, as shown in Table 3. The two industries are situated in the systems of the sports industry and tourism industry, respectively. The two subsystems are connected and coordinated in some way via their interrelationship. From a loosely connected condition in 2014 to a fully coupled state in 2019, China’s sports business and tourism have become more intertwined and coordinated. There is still a lot of opportunity for growth and promise in the industry, which is now operating at a medium level.

### 4.4 Countermeasures for the coupling development of sports industry and tourism

1. Uphold government leadership and improve collaboration between schools and businesses
  The sports bureau and the tourism bureau are two ministries that oversee the sports and tourism sectors, respectively. Therefore, it is crucial that the government continue to take the lead in the process of fusing the sports industry with tourism. Both ministries are particularly concerned in developing and disseminating policies and documents. It is more practical to link when the two departments are coordinated and working together, which is crucial for the relationship between the sports business and tourism. To improve communication and collaboration between the two departments, cooperation should be made more efficient.
2. Improve industrial coupling policy formulation and system construction
  Systematic policy formulation requires the formulation of policies that are in line with the connection between the regional sports industry and tourism based on China’s national conditions and reality, rather than the development of specific industries. It is necessary to realize industrial connection to prevent the protection policy of developing regional characteristics from affecting the connection process. The entire region must take everything into account, enhance the policy system, and improve the management and operation system based on the aforementioned factors and actions. On the other side, the ability of the government to exercise macro-control is also involved.
3. Combine resources and develop brand attributes
  The development of the sports industry and the tourism business have coincided as a result of this combination. When the two industries are just beginning to converge, it is necessary to expand the shortcomings in the branding process by absorbing the cultural essence of the two industries. On the other hand, the coupling of ethnic minority folk culture and local characteristics forms a wide range of industrial connection channels, which enhances the basic competitiveness of the coupled development of sports and tourism industries.

## 5. Conclusions

Sports tourism has grown in popularity as a result of the ongoing rise in human living conditions. It is crucial that people focus on innovation in order to meet the growing demand for sports tourism. Positive and negative coupling are the two categories of which are used to describe industrial coupling. The tourist and sports industries are interconnected in complimentary ways. Between the two, there is no product or technological competition. It has a good effect since it is positively related. This study employs the grey relational analysis approach to assess the relationship and create a connection model in order to demonstrate the unavoidable connection between the two industries. The experiment examines the growth of the sports and tourist sectors in China in recent years. It has been determined that the recent rapid growth of these two industries has boosted local economic growth and employment rates, which has led to the establishment of a more appropriate and scientific index system for evaluating the relationship between the sports industry and the tourism industry. The existing index system for assessing the correlation between the sports sector and the tourism industry cannot accurately reflect the most recent trends due to the difficulties in gathering the most recent data from the sports industry. The most recent data needs to be looked into and explored in more detail in the upcoming issue.
